# The DPC4/SMAD4 genetic status determines recurrence patterns and treatment outcomes in resected pancreatic ductal adenocarcinoma: A prospective cohort study

**DOI:** 10.18632/oncotarget.14901

**Published:** 2017-01-30

**Authors:** Sang Hyun Shin, Hwa Jung Kim, Dae Wook Hwang, Jae Hoon Lee, Ki Byung Song, Eunsung Jun, In Kyong Shim, Seung-Mo Hong, Hyoung Jung Kim, Kwang-Min Park, Young-Joo Lee, Song Cheol Kim

**Affiliations:** ^1^ Division of Hepato-Biliary and Pancreatic Surgery, Department of Surgery, Asan Medical Center, University of Ulsan College of Medicine, Seoul, South Korea; ^2^ Department of Preventive Medicine, University of Ulsan College of Medicine, Seoul, South Korea; ^3^ Department of Biomedical Sciences, University of Ulsan College of Medicine, Seoul, South Korea; ^4^ Biomedical Research Center, Asan Institute for Life Sciences, Asan Medical Center, Seoul, South Korea; ^5^ Department of Pathology, Asan Medical Center, University of Ulsan College of Medicine, Seoul, South Korea; ^6^ Department of Radiology and the Research Institute of Radiology, Asan Medical Center, University of Ulsan College of Medicine, Seoul, South Korea

**Keywords:** pancreatic ductal adenocarcinoma, pancreatic cancer, DPC4, SMAD4

## Abstract

**Objectives:**

The objective of this study was to investigate the role of genetic status of *DPC4* in recurrence patterns of resected pancreatic ductal adenocarcinoma (PDAC).

**Methods:**

Between April 2004 and December 2011, data on patients undergoing surgical resection for PDAC were reviewed. Genetic status of *DPC4* was determined and correlated to recurrence patterns and clinical outcomes.

**Results:**

Analysis of 641 patients revealed that genetic status of *DPC4* was associated with overall survival and was highly correlated with recurrence patterns, as inactivation of the *DPC4* gene was the strongest predictor of metastatic recurrence (odds ratio = 4.28). Treatment modalities for recurrent PDAC included chemotherapy alone and concurrent chemotherapy along with local control. For both locoregional and metastatic recurrence, local control resulted in improved survival; however, for groups subdivided according to recurrence patterns and genetic status of *DPC4*, local control contributed to improved survival in locoregional recurrences of patients with expressed *DPC4*, while chemotherapy alone was sufficient for others.

**Conclusions:**

Genetic status of *DPC4* contributes to the recurrence patterns following pancreatectomy, and patients with an initially expressed *DPC4* gene receive a greater benefit from intensive local control for locoregional recurrence. The *DPC4* gene, therefore, may aid the establishment of treatment strategies for initial adjuvant treatment or for recurrent PDAC.

## INTRODUCTION

Advances in understanding the molecular underpinnings of pancreatic ductal adenocarcinoma (PDAC) have begun to contribute to the development of new approaches to clinical management of this devastating cancer. Although these studies are in their infancy, preliminary findings have supported the efficacy of molecular approaches for treatment of PDAC. The development and growth of PDAC involves various genetic alterations in oncogenic activation, loss of tumor suppressor gene function, and the overexpression of receptor-ligand systems. Among the several key genes known to contribute to pancreatic carcinogenesis, genetic alterations in *K-ras* and *DPC4/SMAD4* are correlated with patient survival [1–5]. Mutational subtypes of the *K-ras* oncogene have been previously studied for targeted genetic therapy in various cancers, including pancreatic cancer [6–9].

The *DPC4* gene, which is inactivated in 55-80% of PDACs, is one of the major tumor suppressor genes targeted in infiltrating PDAC [4, 10–12]. Loss of *DPC4* expression occurs late in neoplastic progression and leads to the development of infiltrating pancreatic cancer at the stage of histologically recognizable carcinoma. *DPC4* loss also appears to be associated with tumor progression, patterns of failure, and the epithelial-to-mesenchymal transition [10, 13]. The novel study by Iacobuzio–Donahue *et al*. demonstrated that the genetic status of *DPC4* was correlated with patterns of failure in patients with pancreatic cancer [14]. These investigators performed rapid autopsies on patients with documented pancreatic cancer and found that the histological features and patterns of failure were correlated with the genetic status of *DPC4* (i.e., locally destructive tumors in patients with an expressed *DPC4* gene vs. distant metastasis in patients with an inactivated *DPC4* gene). Based on these findings, Iacobuzio–Donahue and colleagues concluded that determination of the status of *DPC4* upon initial diagnosis may aid the stratification of patients into treatment regimens related to local control versus systemic therapy; however, further follow-up prospective studies designed to confirm and extend this finding were proposed.

In terms of treatment, even after curative resection of PDAC, the recurrence rate is very high at early stages, and no effective therapeutic strategies for the treatment of recurrent PDAC have been established to date. To advance the current therapeutic strategies, it is important to determine the factors or treatment modalities that affect prognosis after PDAC recurrence. Therefore, based on the novel findings of Iacobuzio-Donahue *et al*, we hypothesized that the efficacy of the treatment modality for recurrent PDAC may be closely associated with the biological features of the *DPC4* gene. To assess the relationship between the *DPC4* gene and both recurrence and treatment, we prospectively collected patient data regarding the initial *DPC4* genetic status of PDAC. We reviewed recurrence patterns and responses to treatment modalities according to the genetic status of *DPC4*. The results of this study indicate that the genetic status of *DPC4* plays a key role in the recurrence patterns following pancreatectomy for PDAC and can be used in the establishment of therapeutic strategies for recurrent PDAC.

## RESULTS

### Study population

Clinicopathological features of the study cohort are listed in Table 1. The 641 patients included 374 men and 267 women with a median age at diagnosis of 61.0 years (range: 22.0–84.0). There were 198 (30.9%) patients with a preoperative history of diabetes mellitus (DM). The preoperative CA19-9 levels were elevated in 405 (63.2%) patients. Patients had disease in the head/uncinate process of the pancreas (61.8%), the body/tail of the pancreas (26.8%), and the entire pancreas (11.4%). Pathological reports described 7 (1.1%) patients with T1 stage disease, 22 (3.4%) patients with T2, 601 (93.8%) patients with T3, and 11 (1.7%) patients with T4. Also, 280 (43.7%) patients had N0 stage disease, and 361 (56.3%) patients had N1 stage disease. Combined major vascular resection was performed in 183 (28.5%) patients. R0 resection was achieved in 548 (85.5%) patients. Most of the tumors were moderately differentiated (74.3%), while12.6% were poorly differentiated and 10.5% were well differentiated. Perineural invasion was present in 516 (80.5%) patients, while lymphovascular invasion was present in 266 (41.5%) patients. The *DPC4* gene was inactivated in 68.1% of the study subjects. After pancreatectomy, 374 (58.3%) and 72 (11.2%) patients received adjuvant chemotherapy or concurrent chemoradiotherapy, respectively. Table 1 lists the median survival and statistical significance values according to each clinicopathological factor. The overall survival (OS) of patients was significantly associated with the following factors: CA19-9 level, cancer location, T stage, N stage, major vessel resection, resection margin status, tumor differentiation, presence of perineural invasion, presence of lymphovascular invasion, inactivation of the *DPC4* gene, and adjuvant therapy.

**Table 1:** Clinicopathological features and survival analysis of all resected pancreatic ductal adenocarcinoma (n=641)

Variables	n	%	Median survival (months)	*p*
**Clinical factors**				
Sex				
Male	374	58.3	21.9	
Female	267	41.7	19.0	0.84
Age, years				
Median	61.0			0.15
Range	22 - 84			
Preoperative DM				
No	443	69.1	21.6	
Yes	198	30.9	20.0	0.61
CA 19-9^†^				
Normal (≤37)	226	35.3	28.2	
Elevated (>37)	405	63.2	18.0	< 0.001
NA	10	1.5		
**Tumor factors**				
Location of cancer				
Head/Uncinate process	396	61.8	20.1	
Body/Tail	172	26.8	31.7	0.04
Entire pancreas	73	11.4	11.5	< 0.001
T stage				
T1	7	1.1	NA	
T2	22	3.4	34.3	0.15
T3	601	93.8	20.5	0.01
T4	11	1.7	22.3	0.16
N stage				
N0	280	43.7	30.0	
N1	361	56.3	17.4	< 0.001
Major vessel resection				
No	458	71.5	24.3	
Yes	183	28.5	15.2	< 0.001
Resection margin status				
R0	548	85.5	21.7	
R1	93	14.5	16.4	0.02
Differentiation^†^				
WD	67	10.5	33.4	
MD	476	74.3	20.5	0.03
PD	81	12.6	12.5	< 0.001
NA	17	2.6		
Perineural invasion				
Absent	125	19.5	28.2	
Present	516	80.5	19.5	0.002
Lymphovascular invasion				
Absent	375	58.5	25.1	
Present	266	41.5	15.7	< 0.001
DPC4 gene				
Normal	165	25.7	25.4	
Inactivated	476	74.3	19.4	0.03
**Adjuvant therapy**				
No	195	30.4	16.6	
CTx alone	374	58.3	22.7	0.006
CCRTx	72	11.2	28.2	0.004

### Analysis of recurrence patterns

Linear logistic regression analysis was conducted to identify factors that affected recurrence patterns (Table 2). Localization throughout the entire pancreas, inactivation of *DPC4* gene function, and no adjuvant therapy were identified as independent factors that determined metastatic recurrence. Among these factors, inactivation of the *DPC4* gene was the most strongly correlated with metastatic recurrence (adjusted odds ratio, [aOR] = 4.28).

**Table 2:** Linear logistic regression identifying factors affecting metastatic recurrence in resected pancreatic ductal adenocarcinoma

Variables	uOR	95% CI	aOR	95% CI
***Clinical factors***				
Sex				
Male	Reference			
Female	0.83	0.57 to 1.23		
Preoperative DM				
No	Reference			
Yes	1.14	0.76 to 1.73		
CA 19-9				
Normal (≤37)	Reference			
Elevated (>37)	0.88	0.58 to 1.33		
***Tumor factors***				
Location of cancer				
Head/Uncinate process	Reference		Reference	
Body/Tail	1.50	0.95 to 2.37	1.75	**1.07 to 2.86**
Entire pancreas	1.82	1.21 to 4.44	2.06	**1.04 to 4.07**
T stage				
T1/2	Reference			
T3/4	1.22	0.44 to 3.37		
N stage				
N0	Reference			
N1	0.98	0.67 to 1.45		
Major vessel resection				
No	Reference			
Yes	1.11	0.73 to 1.68		
Resection margin status				
R0	Reference			
R1	1.02	0.59 to 1.73		
Differentiation				
WD	Reference			
MD	1.43	0.76 to 2.69		
PD	2.21	0.98 to 4.98		
Perineural invasion				
Absent	Reference			
Present	1.03	0.63 to 1.68		
Lymphovascular invasion				
Absent	Reference			
Present	1.06	0.72 to 1.55		
*DPC4* gene				
Normal	Reference		Reference	
Inactivated	**4.21**	**2.74 to 6.48**	**4.28**	**2.75 to 6.68**
***Adjuvant therapy***				
No	Reference		Reference	
CTx alone	**0.58**	**0.37 to 0.92**	**0.51**	**0.32 to 0.84**
CCRTx	0.73	0.36 to 1.48	0.82	0.38 to 1.74

uOR, unadjusted odds ratio; aOR, adjusted odds ratio; DM, diabetes mellitus; WD, well differentiated; MD, moderately differentiated; PD, poorly differentiated; CTx, chemotherapy; CCRTx, concurrent chemo-radiotherapy.

### DPC4 gene status defines infiltrative or metastatic behavior and affects patient prognoses

Throughout the study cohort, computed tomography (CT) images of patients obtained between January 2011 and December 2011 were reviewed by a radiologist (Hyoung Jung Kim) at our institute. Peripancreatic infiltration was defined as peritumoral fatty stranding, and vascular invasion was evaluated by using the criteria of tumor thrombus, vessel occlusion, stenosis and contour deformity [15, 16]. CT imaging characteristics were compared according to the genetic status of *DPC4* (Table 3). Expressed *DPC4* (*DPC4*+) cancers tended to be well-defined with less peripancreatic infiltration compared to inactivated *DPC4* (*DPC4*-) cancers (81.3% vs. 94.3%, *p*=0.01); however, major arterial or venous invasions did not differ between the two groups.

**Table 3:** Correlation of CT imaging characteristics with *DPC4* gene status in the patients diagnosed as pancreatic ductal adenocarcinoma between January 2011 and December 2011

Variables		*DPC4*+(n=75)	*DPC4*-(n=88)	*p*
Peripancreatic infiltration	Present	61 (81.3%)	83 (94.3%)	**0.01**
	Absent	14 (18.7%)	5 (5.7%)	
Artery invasion	Present	23 (30.7%)	35 (39.8%)	0.23
	Absent	52 (69.3%)	53 (60.2%)	
Vein invasion	Present	23 (30.7%)	32 (36.4%)	0.44
	Absent	52 (69.3%)	56 (63.6%)	

**Figure 1:**
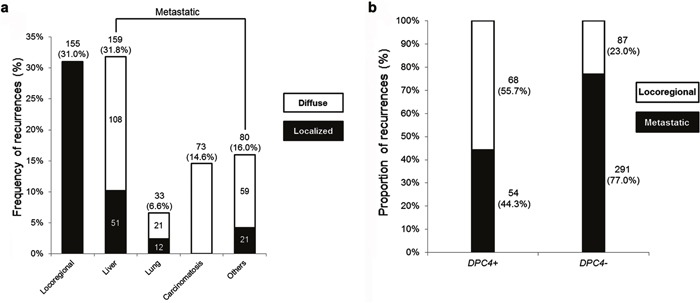
Diagrams for recurrence patterns of overall patients and proportion of recurrences according to the *DPC4* status **a**. Frequency of recurrence following pancreatectomy for pancreatic ductal adenocarcinoma (PDAC). The liver was the most common site of metastasis. **b**. Proportion of recurrences stratified by *DPC4* gene status. The study cohort consisted of 500 patients with recurrent disease in 68 locoregional and 54 metastatic recurrences in the *DPC4+* group and in 87 locoregional and 291 metastatic recurrences in the *DPC4-* group.

Among the 641 resected PDAC patients, 165 (25.7%) and 476 (74.3%) patients had expressed and inactivated *DPC4* genes, respectively. During the follow-up period, 500 patients had recurrent disease, including 155 locoregional and 345 metastatic recurrences. Metastatic recurrences of overall patients could be subdivided into localized or diffuse metastases (Figure 1A). The proportion of metastatic recurrence was significantly higher in the *DPC4*- than in the *DPC4*+ patients. The most common metastatic site was the liver (31.8% of all recurrences), and 51 patients had localized hepatic metastasis. The “others” (16.0%) included the para-aortic lymph nodes, intestines, and other tissues. According to *DPC4* status, there were 68 locoregional and 54 metastatic recurrences in the *DPC4*+ group, and 87 locoregional and 291 metastatic recurrences in the *DPC4*- group (Figure 1B). Table 4 shows initial sites of recurrence and detailed treatment modalities according to the *DPC4* gene status.

**Table 4:** Initial sites of recurrence and detailed treatment modalities according to the *DPC4* gene status (n = 500)

c			Treatment for recurrent disease						
			Chemotherapy + Local control					Chemotherapy alone	No therapy
Initial site of recurrence	n	%	Operation	RFA	TACI	TACE	RTx		
DPC4+									
Locoregional	68	13.6	5	-	-	-	7	39	17
Metastatic									
Liver	30	6.0	2	5	-	-	2	13	8
Lung	7	1.4	1	-	-	-	-	4	2
Peritoneal carcinomatosis	9	1.8	-	-	-	-	-	6	3
Others	8	1.6	-	-	-	-	3	2	3
DPC4-									
Locoregional	87	17.4	4	-	-	-	12	46	25
Metastatic									
Liver	129	25.8	4	16	1	1	2	70	35
Lung	26	5.2	5	-	-	-	2	14	5
Peritoneal carcinomatosis	64	12.8	-	-	-	-	-	36	28
Others	72	14.4	2	-	-	-	8	34	28

The *DPC4*+ and *DPC4*- groups were also assessed following restriction of the study population to 500 patients with recurrent PDAC (Table 5). Cancer located throughout the pancreas was ~3-fold more frequent in the *DPC4*- group (16.1%) than in the *DPC4*+ group (5.7%), while metastatic recurrence patterns were more dominant in the *DPC4*- group (77.0%) than in the *DPC4*+ group (44.3%). Furthermore, the cancer location (*p*=0.01) and resection margin status (*p*=0.05) were each associated with *DPC4* gene status.

**Table 5:** Correlation between clinicopathological features and the status of *DPC4* gene in patients with recurrent cancer (n=500)

Variables	*DPC4*+ (%)	*DPC4*- (%)	*p*
***Recurrence patterns***			**< 0.001**
Locoregional	68 (55.7)	87 (23.0)	
Metastatic	54 (44.3)	291 (77.0)	
***Clinical factors***			
Sex			0.99
Male	73 (59.8)	226 (59.8)	
Female	49 (40.2)	152 (40.2)	
Age, years			0.72
Mean ± SD	59.6 ±9.3	60.0 ±10.2	
Preoperative DM			0.53
Absent	35 (28.7)	120 (31.7)	
Present	87 (71.3)	258 (68.3)	
CA19-9 (n=491)			0.87
Normal (≤ 37U/mL)	39 (32.0)	115 (31.2)	
Elevated (> 37 U/mL)	83 (68.0)	254 (68.8)	
***Tumor factors***			
Location of cancer			**0.01**
Head/Uncinate process	79 (64.8)	226 (59.8)	
Body/Tail	36 (29.5)	91 (24.1)	
Entire pancreas	7 (5.7)	61 (16.1)	
T stage			0.597†
T1/2	4 (3.3)	13 (3.4)	
T3/4	118 (96.7)	365 (96.6)	
N stage			0.93
N0	48 (39.3)	147 (38.9)	
N1	74 (60.7)	231 (61.1)	
Major vessel resection			0.264
No	89 (73.0)	255 (67.5)	
Yes	33 (27.0)	123 (32.5)	
Resection margin status			**0.05**
R0 resection	97 (79.5)	328 (86.8)	
R1 resection	25 (20.5)	50 (13.2)	
Differentiation (n=488)			0.17
WD	16 (13.7)	30 (8.1)	
MD	83 (70.9)	288 (77.6)	
PD	18 (15.4)	53 (14.3)	
Perineural invasion			0.15
Absent	27 (22.1)	62 (16.4)	
Present	95 (77.9)	316 (83.6)	
Lymphovascular invasion			0.48
Absent	64 (52.5)	212 (56.1)	
Present	58 (47.5)	166 (43.9)	
***Adjuvant therapy***			
No	31 (25.4)	109 (28.8)	0.12
CTx alone	72 (59.0)	234 (61.9)	
CCRTx	19 (15.6)	35 (9.3)	

^†^, Fisher's exact test;

SD, standard deviation; DM, diabetes mellitus; WD, well differentiated; MD, moderately differentiated; PD, poorly differentiated; CTx, chemotherapy; CCRTx, concurrent chemo-radiotherapy.

**Figure 2:**
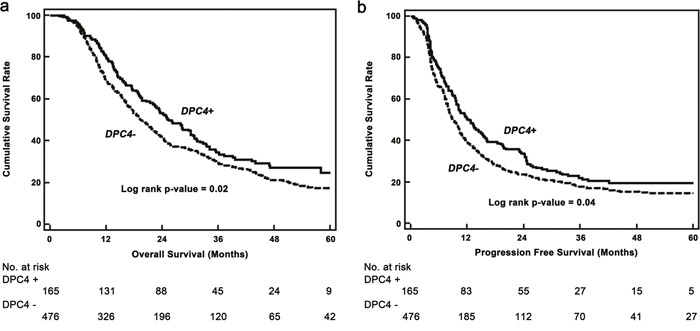
Kaplan–Meier survival curves **a**. Overall survival based on the genetic status of *DPC4* (n=641). Median overall survival of patients with *DPC+* and *DPC-* cancers were 25.4 and 19.4 months (*p*=0.02), respectively. **b**. Progression-free survival based on the genetic status of *DPC4*. The median progression free survival of *DPC4+* and *DPC4-* cancers were 11.4 and 8.9 months (*p*=0.04), respectively.

The OS and progression free survival (PFS) of patients were also evaluated based on the genetic status of *DPC4* (Figure 2). The median OS was 25.4 and 19.4 months in the *DPC4*+ and *DPC4*- groups, respectively (*p*=0.02), and the median PFS was 11.4 and 8.9 months in the *DPC4*+ and *DPC4*- groups, respectively (*p*=0.04).

### Concurrent local control for recurrent PDAC enhances patient survival only in DPC4+ cancers

As recurrence patterns are directly associated with treatment strategies, survival after recurrence was compared according to the treatment modality in each recurrence group (Table 6). The application of both chemotherapy (CTx) and local control (LCx) was most effective throughout the entire population: the unadjusted HRs were 0.33 (95% C.I. 0.20-0.57) in the locoregional group and 0.49 (95% C.I. 0.32-0.75) in the metastatic group compared with the untreated group. After adjusting for confounders, CTx was found to be better than no therapy, and the addition of a local control improved survival, irrespective of recurrence patterns. This phenomenon was consistent, even in comparisons between CTx alone and CTx + LCx in both recurrence groups (*p*=0.004, locoregional; *p*=0.04, metastatic).

**Table 6:** Analysis for survival after recurrence according to the recurrence patterns and treatment modalities (n=500)

Variables	uHR	95% CI	*p* for trend	aHR^†^	95% CI	*p* for trend	*p* for CTx alone vs. CTx+LCx
Locoregional recurrence							
No therapy	Reference		**< 0.001**	Reference		**< 0.001**	
CTx alone	**0.61**	**0.42 to 0.90**		**0.60**	**0.41 to 0.88**		**0.004**
CTx + LCx	**0.33**	**0.20 to 0.57**		**0.35**	**0.20 to 0.59**		
Metastatic recurrence							
No therapy	1.23	0.86 to 1.77	**< 0.001**	1.19	0.82 to 1.71	**< 0.001**	
CTx alone	0.72	0.51 to 1.01		**0.68**	**0.48 to 0.96**		**0.04**
CTx + LCx	**0.49**	**0.32 to 0.75**		**0.51**	**0.34 to 0.78**		

**Figure 3:**
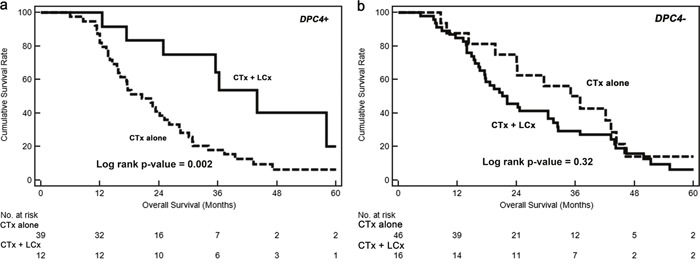
Kaplan–Meier survival curves for locoregional recurrences **a**. The median overall survival of concurrent chemotherapy and local control (CTx + LCx) versus chemotherapy alone (CTx alone) in *DPC4+* cancers were 44.0 versus 20.5 months (*p* = 0.002), respectively. **b**. The median overall survival of CTx + LCx versus CTx only were 21.1 versus 35.1 months (*p* = 0.32), respectively.

To probe the effects of treatment according to *DPC4* genetic status, we investigated the correlations between overall survival in each subgroup subdivided by treatment modalities, recurrence patterns, and the *DPC4* gene status (Table 7). In all subgroups, the addition of LCx improved the OS. Notably, the unadjusted hazard ratio (uHR) of CTx + LCx for locoregional recurrence in the *DPC4*+ subgroup was 0.25 (95% C.I. 0.10-0.61) and the aHR was 0.24 (95% C.I. 0.10-0.59) after adjusting for confounders. Comparisons between CTx alone and CTx + LCx revealed that the addition of local control for locoregional recurrence in the *DPC4*+ group, but not in the *DPC4-* group, yielded the greatest benefit (*p*=0.002) in improving survival. Figure 3 also shows survival benefit of local control for locoregional recurrence in *DPC4*+ group. Therefore, the effects of treatment modalities could be influenced by recurrence patterns according to the genetic status of *DPC4*.

**Table 7:** Analysis for overall survival according to the recurrence patterns and treatment modalities in relations with the *DPC4* gene status (n=500)

Variables	uHR	95% CI	*p* for trend	aHR^†^	95% CI	*p* for trend	*p* for CTx alone vs. CTx+LCx
DPC4+							
Locoregional recurrence							
No therapy	Reference		**< 0.001**	Reference		**< 0.001**	
CTx alone	0.73	0.41 to 1.31		0.68	0.37 to 1.22		**0.002**
CTx + LCx	**0.25**	**0.10 to 0.61**		**0.24**	**0.10 to 0.59**		
Metastatic recurrence							
No therapy	2.04	1.01 to 4.13	**< 0.001**	1.99	0.97 to 4.06	**< 0.001**	
CTx alone	0.75	0.40 to 1.42		0.80	0.42 to 1.12		0.12
CTx + LCx	**0.39**	**0.17 to 0.88**		**0.38**	**0.17 to 0.89**		
DPC4-							
Locoregional recurrence							
No therapy	0.94	0.50 to 1.76	**0.01**	0.85	0.45 to 1.60	**< 0.001**	
CTx alone	0.59	0.33 to 1.05		**0.53**	**0.30 to 0.95**		0.32
CTx + LCx	**0.44**	**0.21 to 0.89**		**0.39**	**0.19 to 0.81**		
Metastatic recurrence							
No therapy	1.28	0.75 to 2.17	**0.01**	1.17	0.69 to 2.00	**< 0.001**	
CTx alone	0.83	0.49 to 1.39		0.68	0.40 to 1.15		0.12
CTx + LCx	**0.55**	**0.31 to 0.99**		**0.53**	**0.29 to 0.96**		

## DISCUSSION

Without surgical resection, PDAC is often incurable. Even after surgery, early recurrence or metastasis frequently occur, and the overall survival rate remains low. These characteristics usually discourage efforts to treat this disease. As shown by the current findings, most of the clinicopathological features of patients with PDAC result in a dismal prognosis. Although adjuvant therapies, including CTx and/or radiotherapy, have shown the potential to prevent or cure disease, many limitations and failures of these therapies are evident in the observation that 500 (78.0%) of 641 patients exhibited recurrent cancer during our study period. Accordingly, we should focus our attention on treatments for both recurrent and primary PDAC.

In this current era of molecular biology, a better understanding of the cellular and molecular features of cancer may yield major advances in its clinical management. In the present study, we investigated correlations between the genetic status of *DPC4* and the postoperative clinical course in a large cohort of PDAC cases. Furthermore, we demonstrated the importance of the *DPC4* gene in tumor progression following surgical resection and investigated treatment status for recurrent cancer in correlation with the *DPC4* status.

In agreement with these results, several reports [2–4, 17, 18] have shown that *DPC4* gene status was associated with patient prognoses; however, no direct correlation at the molecular level has yet been established. *SMAD4 (DPC4)* plays an important role in both tumor suppression and progression [19]. Sustained exposure to the cytokine transforming growth factor-β (TGF-β), which leads to the epithelial-to-mesenchymal transition (EMT) and the inhibition of growth arrest and apoptosis, suppresses Smad signaling [20]. Additionally, the Smad proteins play a central role in TGF-β-dependent EMT associated with tumor progression and metastasis [21]. Yamada *et al* reported that patients with epithelial tumors had a better OS than mesenchymal-type tumors, which often lack DPC4 expression, and showed that EMT was the most significant independent prognostic factor for pancreatic cancer [13, 22].

**Figure 4:**
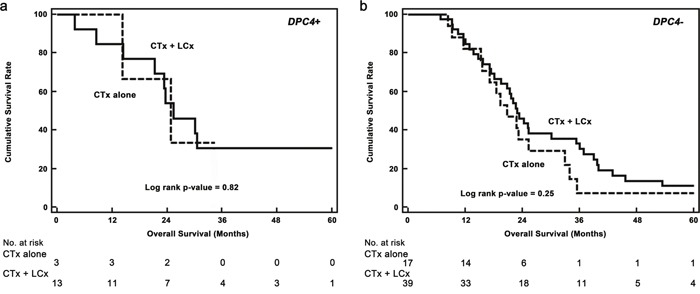
Kaplan–Meier survival curves for potentially resectable (localized) metastatic recurrences **a**. The median overall survival of concurrent chemotherapy and local control (CTx + LCx) versus chemotherapy alone (CTx alone) in *DPC4+* cancers were 23.6 versus 30.1 months (*p* = 0.82), respectively. **b**. The median overall survival of CTx + LCx versus CTx only were 22.8 versus 20.7 months (*p* = 0.25), respectively.

**Figure 5:**
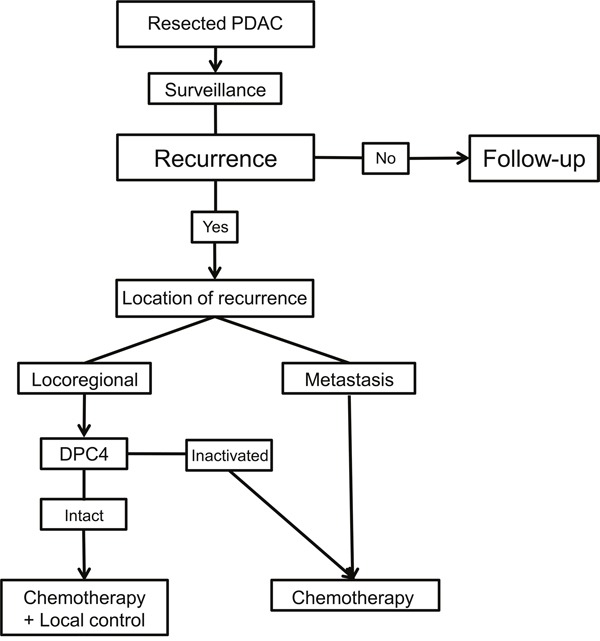
Suggested treatment algorithm for the recurrence of resected pancreatic ductal adenocarcinoma (PDAC) during surveillance

In a clinical setting, Iacobuzio–Donahue *et al* [14] reported that the initial *DPC4* genetic status in PDAC was correlated with patterns of failure, which were locally destructive or metastatic tumors; however, these investigators concluded that further follow-up prospective studies were needed. Our present study also revealed that the genetic status of *DPC4* highly reflected clinical features and initial recurrence patterns following pancreatectomy: an expressed *DPC4* gene was associated with locoregional recurrence, and inactivation of *DPC4* was correlated with metastatic recurrence. At our institute, we performed repeated resections or locally targeted treatments (radiofrequency ablation, transarterial chemoembolization or radiotherapy) with the consent of patients if the lesion was confined to a locoregional area or was metastatic. The feasibility of repeated resection for recurrent PDAC after initial pancreatectomy is not yet accepted; however, several previous studies [23–25] support the concept of repeated local therapy for either locoregional or metastatic recurrences. Analysis of the effects of intensive local therapy, including repeated resection, ablation, and radiotherapy, for recurrent PDAC indicated that the application of both CTx and LCx was most effective followed by CTx alone. The gradient of survival risk was precipitous in the locoregional recurrence group compared with the metastatic group. Although systemic CTx is a well-established treatment of choice for recurrent PDAC, we found that the addition of intensive local therapy contributes to improved survival. Therefore, diminishing the tumor burden may create a synergistic effect to improve survival in cooperation with systemic CTx. Comparison of OS according to the genetic status of *DPC4* and treatment modalities led to a better understanding of the role of local therapy (Table 7). In this analysis, concurrent local control was found to be effective for locoregional recurrence in *DPC4*+ cancers but not for metastatic recurrence or in *DPC4*- cancers. To calibrate biases caused by differences in the severity of the metastatic burden, we conducted subgroup analysis for potentially resectable (localized) metastasis (Figure 4). The median survival following CTx + LCx and CTx alone for potentially resectable metastases was 23.6 and 30.1 months in *DPC4+* cancers (*p*=0.82), respectively and 22.8 and 20.7 months in *DPC4-* cancers (*p*=0.25), respectively. These findings confirmed our hypothesis that the effect of local control may be maximized in locoregional recurrence with an expressed *DPC4* gene. Based on the correlation of the genetic status of DPC4 with recurrence patterns and the role of local control, we suggest the use of a treatment algorithm for recurrent PDAC during surveillance following pancreatectomy (Figure 5).

A previous study reported that *DPC4* failed to predict recurrence pattern. Winter et al reported in their analysis with 127 resected PDAC that loss of *DPC4* expression was 31.5%, and it was neither associated with recurrence pattern nor associated with early death [26]. However, the loss rate was quite different from other previous reports showing that *DPC4* was lost in 55 up to 80% of PDAC [2, 4, 5, 10, 13, 27, 28]. Although the findings of the present study showed importance of *DPC4* status in recurrence, we acknowledge that the recurrence patterns are not solely affected by *DPC4* status, and there has been still controversy in the prognostic value of *DPC4* status.

This study has selection bias, which is one possible limitation of our study, may have been present in the treatment plans for recurrent PDAC due to differences in prescribed treatment strategies among physicians of various subspecialties, such as surgeons, gastroenterologists, and oncologists. And there were limitations that exact role of each local procedure could not be identified because the number of each LCx was small. In addition, the genetic data have been collected prospectively, but this is still a retrospective review of the cases. Therefore, additional studies will be required to verify our present findings. Nonetheless, as our current analysis was conducted with the largest cohort reported to date at a high-volume center with well-established treatment guidelines, our findings provide significant insight into *DPC4* gene function in terms of recurrence patterns and treatment plans for recurrent PDAC.

In conclusion, our study of more than 500 patients with PDAC examined the correlations between the clinical course of PDAC and the initial genetic status of *DPC4*. Our findings suggest clinical relevance of the genetic status of *DPC4* in terms of distinct features, including infiltrative features and recurrence patterns, as well as responses to treatment modalities. The genetic status of *DPC4* contributes to the recurrence patterns observed follow pancreatectomy for PDAC, and patients with an initially expressed *DPC4* gene receive greater benefits from intensive local control therapy for locoregional recurrence. Therefore, studies of the genetic status of *DPC4* will help to establish treatment strategies for either the adjuvant setting or recurrent PDAC.

## MATERIALS AND METHODS

### Patients

Between April 2004 and December 2011, a total of 689 consecutive patients with PDAC underwent surgical resection at Asan Medical Center (Seoul, South Korea). Patient data, including genetic alterations, were prospectively collected and retrospectively reviewed using electronic medical records available at our institute. This study was approved by our Institutional Review Board, and all genetic studies were performed after obtaining informed consent. Among the 689 PDAC cases, 26 had stage IV disease, 7 died of other causes, and 15 were lost to follow-up. As a result, 641 patients were included in the current analyses. The margin status of resected specimen was reviewed, and R1 was defined as a distance of the tumor from the resection margin of ≤1mm [29, 30].

### Detection of genetic alterations of the DPC4 gene

**Figure 6:**
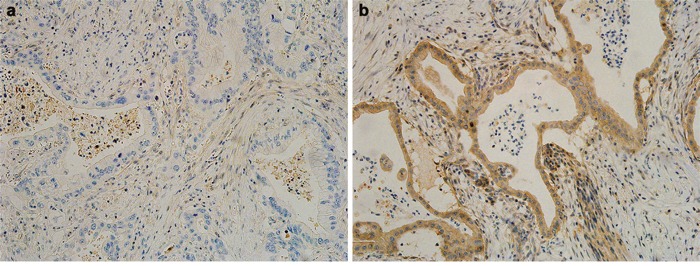
Representative photographs of the immunohistochemistry analysis of DPC4 in pancreatic ductal adenocarcinoma **a**. Negative staining indicates an inactivated *DPC4* gene. **b**. Positive staining indicates an expressed *DPC4* gene. All photographs are shown at a ×200 magnification.

As we described previously [4], the genetic status of *DPC4* was assessed by immunohistochemical staining. After deparaffinization and antigenic retrieval, slides were labeled with a monoclonal antibody to DPC4 (clone EP618Y, diluted 1:100; Abcam Inc., Cambridge, MA, USA). Labeling was achieved using the avidin-biotin complex method. The chromogen 3-amino-9-ethylcarbazole was used. Normal saline was used as a substitute for the primary antibody as a negative control. A single pathologist interpreted and scored the immunohistochemistry staining of *DPC4*. In pathology reports of slides stained using immunohistochemistry, the frequency of *DPC4*-positive cells in a tumor population were scored as 0 to 3 as follows: 0, less than 10%; 1, 10% to 33% positive; 2, 34% to 67% positive; and 3, more than 67% positive. After scoring, cases were dichotomized as intact/decreased DPC4 expression (score 1-3) and total loss of DPC4 expression (score 0). Representative photographs of immunohistochemistry staining are shown in Figure 6. Negative staining indicated an inactivated *DPC4* gene (Figure 6A), and positive staining indicated an expressed *DPC4* gene (Figure 6B).

### Adjuvant therapy, postoperative surveillance, and detection of the primary recurrent site

Postoperative adjuvant treatment was administered between 3 weeks and 3 months after surgery. Patients received either 5-fluorouracil with leucovorin or gemcitabine for 6 months. In patients with microscopic residual disease (R1), 5-fluorouracil-based chemoradiation was added. Contrast-enhanced abdominoperineal CT was used for postoperative surveillance, and CA 19-9 levels were examined every 3 months for the first 2 years following surgery and then every 6 months. Diagnoses of locoregional recurrence, which included the region of the pancreatic bed, the root of the mesentery, and hepatoduodenal ligament, were based on progressive soft tissue growth at specific sites and elevated CA19-9 levels [31]. Metastatic recurrence was defined as recurrence in the peritoneal cavity or other remote organs, including the liver, lung, or other organs. When lesions of potential recurrent disease were detected, ^18^F-fluorodeoxyglucose positron emission tomography (FDG-PET), chest CT, and/or biopsy were performed to confirm the diagnosis of recurrence. Cases with simultaneous locoregional and metastatic recurrences were identified as metastatic recurrence. Metastatic recurrence was sometimes subdivided into localized or diffuse metastases. Localized metastasis indicated metastasized lesion(s) in a focal area, such like a single lobe of liver or lung. Diffuse metastasis indicated lesions throughout multiple areas.

### Treatment modalities for recurrent disease

According to the therapeutic guidelines of our institute, patients with recurrent PDAC are candidates for systemic chemotherapy if the performance status allows. Additionally, aggressive LCx is also employed if the recurrent PDAC is locally controllable. LCx includes complete total pancreatectomy, tumorectomy, hepatic resection, pulmonary resection, radiofrequency ablation, transarterial chemoembolization, and radiotherapy. In the present study, the treatment modalities for recurrence were subdivided into the following three groups: conservative management (no treatment), CTx alone, and concurrent CTx and LCx.

### Statistical analysis

Univariable comparisons of estimated survival according to clinicopathological factors and genetic alterations were performed using the Kaplan-Meier method and log-rank test. A linear logistic regression model was used to identify factors that affected recurrence patterns, and a descriptive analysis was conducted to examine the relationships between the *DPC4* genetic status and either recurrence or survival. Multiple Cox proportional hazard models were used to assess associations between the status of the *DPC4* gene and either recurrence patterns or kinetics according to survival after adjusting for covariates, including clinically important confounders that were selected using statistical analyses. The *p*-value for each trend was calculated by treating the treatment modality group as an ordinal variable (i.e., with three different levels: 0 as no treatment, 1 as CTx alone, and 2 as CTx and LCx) with or without *DPC4* status (i.e., 0 as expressed, and 1 as deletion). All analyses were performed using SAS (v9.3, SAS Institute, Cary, NC), and a *p*-value < 0.05 indicated a statistically significant difference.
